# Prevalence and Associated Risk Factors of Antenatal Depression among Pregnant Women Attending Tertiary Care Hospitals in South India

**DOI:** 10.1155/2022/9127358

**Published:** 2022-11-17

**Authors:** Savitha Prabhu, Shyamala Guruvare, Linu Sara George, Baby S. Nayak, Shreemathi Mayya

**Affiliations:** ^1^Department of Psychiatric (Mental Health) Nursing, Manipal College of Nursing, Manipal Academy of Higher Education, Manipal, India; ^2^Department of Obstetrics and Gynecology, Kasturba Medical College, Manipal Academy of Higher Education, Manipal, India; ^3^Department of Fundamentals of Nursing, Manipal College of Nursing, Manipal Academy of Higher Education, Manipal, India; ^4^Department of Child Health Nursing, Manipal College of Nursing, Manipal Academy of Higher Education, Manipal, India; ^5^Department of Data Science, Prasanna School of Public Health, Manipal Academy of Higher Education, Manipal, India

## Abstract

**Background:**

Antenatal depression (AND) is a common mood disorder that affects both the mother and the child.

**Objective:**

The current study is aimed at identifying the prevalence of antenatal depression and the risk factors associated with it in South Indian pregnant women.

**Materials and Methods:**

The current study was carried out in a tertiary care teaching hospital where pregnancy and postnatal care are offered. In the study, 314 pregnant women who visited the antenatal clinic for their prenatal checkups were included. To diagnose possible depression, Edinburgh Postnatal Depression Scale (EPDS) was used. The chi-square test was applied to determine the association between antenatal depression and various socioeconomic, obstetric, and medical factors. A logistic regression analysis was performed to identify significant confounding variables.

**Results:**

Of the total 314 women, 69 (21.98%) were suffering from possible depression with the mean EPDS score being 10.61 ± 7.48. Women of younger age had greater risks for depression than older women (AOR = 2.01; 95% CI: 0.56-7.20). Maternal age (*χ*^2^ = 0.013, *p* = 0.009) and the presence of health issues during the current pregnancy (*χ*^2^ = 5.18, *p* = 0.023) were the factors significantly associated with antenatal depression.

**Conclusions:**

Clinical efforts should focus on screening antenatal depression, early identification, and effective care, thus preventing progression to postpartum depression and its detrimental effects.

## 1. Introduction

Pregnancy is a time of physical and psychological preparation for birth and parenthood [1]. Although pregnancy is a joyous moment, it can be recognized as a period of confusion, stress, fear, anxiety, and mood swings for some women [2]. Antenatal depression (AND) is a rising public health issue that has detrimental effects on the health and welfare of women and their families [3]. It can be accompanied by signs and symptoms of sadness, lack of energy, insomnia, weight loss, decreased physical and cognitive functioning, loss of appetite, irritability, and hopelessness [4]. Depression during pregnancy is one of the strongest predictors of postnatal depression, which is in turn a major predictor of mental problems [5]. The general public may also be unaware that two-thirds of postnatal depression cases start before the baby is born, often during the third trimester of pregnancy [6].

Systematic reviews conducted in low- and middle-income countries reported the prevalence of antenatal depression ranging between 19.2% and 34% with variation in sociodemographic, obstetric, and measurement factors [4, 5, 7, 8]. Previous studies in India have reported a wide-ranging prevalence of antenatal depression between 8.7% and 65% [9–13], with variation according to the type of screening tool and study setting. Subsequently, another systematic review in India reported that the prevalence of prenatal depression ranged from 9.18% to 65.0% in Northern, Western, and Southern India [14]. The prevalence rate of 8.8% and 18.5% perinatal depression was reported at community and medical facilities in India [3].

Perinatal depression is associated with adverse perinatal outcomes including preterm birth and low birth weight [15, 16]. Major depression is also relatively common in pregnancy characterized by the idea of suicide or self-harming [6]. Antenatal depression is highly associated with several sociodemographic and obstetric variables. Unplanned pregnancy; strained relationships with in-laws; physical illness [17]; history of abortions; preference for male gender [18, 19]; alcoholism in a spouse; low education status of women [20]; multiple births; younger maternal age [10]; unplanned pregnancy family discord [12]; late pregnancy and lower or middle socioeconomic status [21]; poor/satisfactory relationship with spouse, siblings, or in-laws [22]; domestic violence; worry over the pregnancy; and history of traumatic experiences [23] were all significant predictors of depression in pregnant women.

Perinatal mental health has not received much attention in low- and middle-income nations. Antenatal depression has been underdiagnosed and frequently ignored worldwide, particularly in developing countries like India [12]. Maternal and child health (MCH) programs in India typically place more emphasis on enhancing the nutritional status and maternal weight gain and place less emphasis on a woman's emotional and mental health during the perinatal period. This may be because the physical well-being of the expectant mother receives more attention than her mental health [6, 24]. Moreover, women with antenatal depression are less likely to have frequent prenatal checkups [25].

There is evidence to suggest that interventions in women at risk of antenatal depression were effective in preventing postnatal depression and also improving pregnancy outcomes [26–28]. Subsequently, several systematic reviews and meta-analyses substantiated the above observation [29–31].

The incidence of depression and unfavorable pregnancy outcomes may be reduced by early identification of probable depression in pregnant women. Prenatal depression should be screened for, diagnosed as part of routine pregnancy care, and treated promptly to protect the health and safety of expectant mothers [13]. If it is not treated and recognized promptly, it could develop into postpartum depression [24]. Therefore, prenatal screening is necessary to identify hidden instances and protect mothers from the harsh consequences of postpartum depression. Given this context, the current study is aimed at determining the prevalence of antenatal depression and its associated risk factors among pregnant women in the selected tertiary care hospital in South India.

## 2. Materials and Methods

### 2.1. Study Setting

The cross-sectional study was conducted in the Department of Obstetrics and Gynecology of a selected tertiary care teaching hospital in South India. The Department of Obstetrics and Gynecology is a multispecialty department providing high-quality patient care in antenatal services, postnatal welfare, infertility, oncology, and urogynecology. It consisted of an outpatient department with a daily attendance of around 100 antenatal and postpartum mothers. The bed strength of the hospital is 2032 with an 80% patient occupancy rate and adequate staffing.

### 2.2. Sampling Method

The research was the first phase of the intervention study whose research protocol had already been published [32]. The consecutive sampling method was used to select the sample who met the eligibility criteria and consented to participate in the study. The study examined the data of 314 pregnant women who had registered in the antenatal clinic and completed the baseline data for the study from July 2019 to October 2020. The eligibility criteria included women above or equal to 18 years of age, with confirmed pregnancies of above six months (more than 26 weeks). We excluded women with high-risk pregnancies and those with a history of mental illnesses requiring medication as part of the research methodology.

### 2.3. Method of Data Collection

During the antenatal visit, information was collected from women in their third trimester of pregnancy. Written informed consent was obtained before data collection. A participant information sheet outlining the study's objectives and methodology was provided to participants who expressed an interest in participating in the study. The information's privacy and confidentiality were assured.

### 2.4. Tools and Techniques

#### 2.4.1. Sociodemographic Proforma

We employed a predesigned semistructured questionnaire with two sections: demographic data and obstetric and medical characteristics. Personal information, such as age, education, occupation, type of family, and monthly family income, was collected. Obstetric and medical characteristics including health problems during the current pregnancy, parity, history of miscarriage, thyroid dysfunction, history of psychiatric disorders, and alcoholism in the spouse were collected from the study participants.

#### 2.4.2. Screening for Antenatal Depression

Edinburgh Postnatal Depression Scale (EPDS) was used to assess possible depression in pregnant mothers. The EPDS is a 10-item self-administered questionnaire, designed to screen women for symptoms of postpartum depression during the perinatal period [33]. The EPDS consists of 10 questions that inquire about the respondent's emotional state and depression symptoms during the previous seven days. In India, it is widely used and validated for evaluating prenatal and postnatal depression [34, 35]. The cutoff score of more than or equal to 13 on EPDS was considered based on the previous study done in South India, which suggested that the cutoff of 13 for the Kannada-speaking women was based on receiver operating curve analysis for their data [36].

### 2.5. Ethical Consideration

Ethical permission was obtained from the Institutional Ethics Committee (IEC 864-2018). Informed written consent was obtained from study participants before the data collection, and they were assured of the confidentiality of the information.

### 2.6. Statistical Analysis

The data was statistically analyzed using the SPSS version 16. Results were expressed using descriptive statistics like frequency and percentages. The chi-square test was used to assess the association between sociodemographic, obstetric, and medical characteristics and the risk of prenatal depression. Logistic regression analysis was done adjusting for confounders and risk factors; *p* < 0.05 was considered statistically significant.

## 3. Results

### 3.1. Sociodemographic Characteristics of the Study Participants

The data in Table 1 shows the frequency distribution of respondents based on sociodemographic characteristics. Of the 314 pregnant women, most of them (44.9%) belonged to the age group of 30-34 years, the mean age of the respondents being 29.46 ± 4.295 years. Over two-thirds of them (64.9%) completed secondary education. However, 64.9% of pregnant women were homemakers. More than half of the respondents (59.2%) belonged to a nuclear family. Nearly 61.46% of the women had a monthly family income of less than 15000 Indian rupees.

### 3.2. Obstetric and Medical Factors

Among 314 pregnant women, 32.1% have reported health problems during their current pregnancy. Few women (10.5%) had thyroid dysfunction. A small portion of participants (1.28%) had reported having a history of psychiatric illness. Alcoholism in a spouse was reported by 11.4% of women. The majority of the women were primigravida (54.4%), and a history of miscarriage was reported by 22.93% of the women (Table 2).

### 3.3. Prevalence of Prenatal Depression among the Pregnant Women

**Figure 1:**
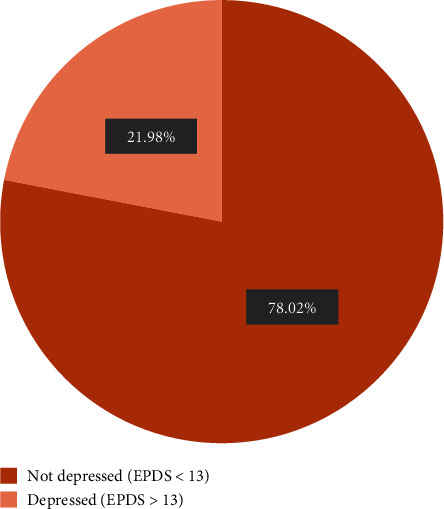
Prevalence of antenatal depression.

Figure 1 reveals that, of the 314 pregnant women, the proportion of those who screened positive for antenatal depression was 22% (69) suggesting a significant likelihood of depression. The mean EPDS score among the participants was 10.61 ± 7.48.

### 3.4. Association of Prenatal Depression with Sociodemographic and Obstetric and Medical Factors (Tables 1 and 2)

Tables 1 and 2 report that age (*χ*^2^ = 11.59, *p* = 0.009) and the presence of health problems during the current pregnancy (*χ*^2^ = 5.18, *p* = 0.023) are associated with prenatal depression on chi-square analysis. There is no association of other sociodemographic and obstetric and medical factors like educational qualification, occupation, type of family, and monthly family income of the respondents with depression (*p* value > 0.05).

In Table 3, the sociodemographic and obstetric factors of women were adjusted for age, educational qualification, occupation of self and husband, and monthly family income. Women with younger age had greater risks for depression than older women (AOR = 2.01; 95% CI: 0.56-7.20); lower education (AOR = 1.52; 95% CI: 0.75-3.05), homemakers than employed (AOR = 1.37; 95% CI: 0.71-2.67), primigravida (AOR = 1.13; 95% CI: 0.33-3.81), and previous history of miscarriage (AOR = 1.56; 95% CI: 0.66-3.70) had a greater risk for antenatal depression. However, the association was not statistically significant.

Overall, age has shown a significant association between univariate (*p* = 0.013) and multivariate regression analysis (*p* = 0.030). Similarly, the presence of health problems also has shown a significant association in univariate analysis (COR = 1.8; 95% CI: 1.0-3.2, *p* = 0.024) with prenatal depression but not in multivariate logistic regression. Multivariate regression analysis of the respondents revealed that there was a five times greater risk of prenatal depression among those with a history of psychiatric illness (AOR = 5.32; 95% CI: 0.58-48.7) shown in Table 3.

## 4. Discussion

Due to the rising tendency of perinatal depression in both developed and developing countries, there is a growing trend for greater awareness of antenatal depression globally. This study examined the prevalence of antenatal depression among pregnant women and its association with several risk factors such as sociodemographic and obstetric and medical characteristics. According to the current study, 69 (22%) of the 314 participants had possible antenatal depression. Research carried out worldwide has revealed significant heterogeneity in the prevalence of prenatal depression. This discrepancy might be explained by socioeconomic status differences and sociocultural and psychosocial aspects. These results concur with those of systematic reviews. A study from South Asia demonstrated 24.3% (95% CI, 19.03 to 30.47) [37], and an Ethiopian study determined 21.28% (95% CI, 15.96–27.78) of the prevalence of prenatal depression [38]. The pooled prevalence of prenatal depression across 173 studies with 182 reports determined was 20.7% (95% CI, 19.4–21.9%, *p* = <0.001, *I*^2^ = 98.4%) [39].

Previous studies from India revealed a significant range in the prevalence of prenatal depression varying from 12.3% to 35.7% [18–21, 24]. This seems to be more in line with our observation of 22%, which represents a marginally higher rate in our study. The timing of the measurement of symptoms throughout the pregnancy, the study setting, the EDPS cutoff score used, the reporting method, the perceptions of mental health, and the study population could all affect the observed variances in our study. Only third-trimester pregnant women were included in our study; during this time, women are more likely to experience mood swings, fatigue, poor sleep, increased worry about childbirth, and other additional responsibilities. This might have increased the number of pregnant women who self-report having depression.

In our study, there was a substantial association between maternal age and prenatal depression. Similar results from some earlier research have been reported [23, 40]. In a Nigerian study, a younger mother's age was also discovered as a risk factor for prenatal depression [41]. Given that, depression frequently manifests in a person's early 20s and that the majority of the study participants were housewives who were financially dependent on their partners. Additionally, the younger pregnant woman, yet in the process of adjusting to the new life in her husband's family, will have an added burden of pregnancy, and depression may be more likely to emerge in this age group.

We observed that the presence of health problems in the current pregnancy was found to be associated with prenatal depression. This has been reported by many other studies as well. A community-based cross-sectional study reported an eightfold increase in perinatal depression in mothers having physical illnesses [19]. Mothers with chronic medical ailments were reported two times more likely to develop antenatal depression [42]. A systematic review examined the association of chronic medical conditions with perinatal mental illnesses [43]. This may be because having health issues by itself reduces people's life satisfaction, which affects their psychological well-being and is one of the potential causes of depression.

## 5. Conclusions

According to this study, prenatal depression has a significant incidence, which evaluated its prevalence and associated factors. The results imply that pregnant women's mental health needs to be given more consideration. In low- and middle-income nations, regular antenatal care should include screening and diagnosis of antenatal depression. Healthcare professionals must create educational programs to assist expectant mothers and offer psychological prenatal care assistance.

## Figures and Tables

**Table 1:** Sociodemographic characteristics of the study sample.

Sl no.	Sociodemographic characteristics	Not depressed (EPDS < 13) (245)	Depressed (EPDS > 13) (69)	Total (314)	Significance
1	*Age in years*	Mean age (*x̅*±SD) = 29.46 ± 4.295			
	20-24	14 (5.71%)	11 (15.94%)	25 (7.96%)	*χ* ^2^ = 11.59 *p* = 0.009^∗^
	25-29	104 (42.44%)	19 (27.53%)	123 (39.17%)	
	30-34	110 (44.89%)	31 (44.92%)	141 (44.90%)	
	35-40	17 (6.93%)	8 (11.59%)	25 (7.96%)	

2	*Education*				
	Below secondary	82 (33.47%)	28 (40.57%)	110 (35.04%)	*χ* ^2^ = 1.19 *p* = 0.274
	Secondary and above	163 (66.53%)	41 (59.42%)	204 (64.96%)	

3	*Occupation*				
	Homemaker	154 (62.85%)	50 (72.47%)	204 (64.97%)	*χ* ^2^ = 2.18 *p* = 0.140
	Employed	91 (37.15%)	19 (27.53%)	120 (38.23)	

4	*Type of family*				
	Nuclear	148 (60.40%)	38 (55.08%)	186 (59.24%)	*χ* ^2^ = 0.635 *p* = 0.426
	Joint	97 (39.60%)	31 (44.92%)	128 (48.76%)	

5	*Monthly family income (in rupees)*				
	Less than 15,000	151 (61.63%)	42 (60.87%)	193 (61.46%)	*χ* ^2^ = 0.013 *p* = 0.908
	15,000 and above	94 (38.37%)	27 (39.13%)	121 (38.54%)	

^∗^Significant.

**Table 2:** Obstetric and medical characteristics of the study sample.

Sl no.	Obstetric and medical characteristics	Not depressed (EPDS < 13) (245)	Depressed (EPDS > 13) (69)	Total (314)	Significance
1	*Presence of health problems during the current pregnancy*				
	Yes	71 (28.98%)	30 (43.47%)	101 (32.17%)	*χ* ^2^ = 5.18 *p* = 0.023^∗^
	No	174 (71.02%)	39 (56.52%)	213 (67.83%)	

2	*Thyroid problem*				
	Yes	25 (10.20%)	8 (11.60%)	33 (10.51%)	*χ* ^2^ = 0.111 *p* = 0.739
	No	220 (89.80%)	61 (88.40%)	281 (89.49%)	

3	*History of past psychiatric illness*				
	Yes	2 (0.82%)	2 (2.90%)	4 (1.28%)	*χ* ^2^ = 1.85 *p* = 0.173
	No	243 (99.18%)	67 (97.10%)	310 (98.72%)	

4	*Alcoholism in spouse*				
	Yes	31 (12.66%)	5 (7.25%)	36 (11.47%)	*χ* ^2^ = 1.55 *p* = 0.213
	No	214 (87.34%)	64 (92.75%)	278 (88.53%)	

5	*Parity*				
	Primigravida	136 (55.52%)	35 (50.72%)	171 (54.46%)	*χ* ^2^ = 0.49 *p* = 0.481
	Multigravida	109 (44.48%)	34 (49.28%)	143 (45.54%)	

6	*Previous history of miscarriage*				
	Yes	52 (21.22%)	20 (28.99%)	72 (22.93%)	*χ* ^2^ = 1.83 *p* = 0.176
	No	193 (78.78%)	49 (71.01%)	242 (77.07%)	

^∗^Significant.

**Table 3:** Bivariate and multivariate analyses of independent variables against antenatal depression.

Variables	Crude odds ratio (COR)	CI (95%)	*p* value	Adjusted odds ratio (AOR)	CI (95%)	*p* value
*Age in years*			0.013^∗^			0.030^∗^
20-24	1.6	0.5 to 5.2	0.384	2.01	0.56 to 7.20	0.279
25-29	0.3	0.1 to 1.0		0.48	0.17 to 1.36	
30-34	0.5	0.2 to 1.5		0.66	0.25 to 1.78	
35-40	Ref			Ref		
*Education*						
Below secondary	0.7	0.4 to 1.2	0.275	1.52	0.75 to 3.05	0.238
Secondary and above	Ref			Ref		
*Occupation*						
Homemaker	1.5	0.8 to 2.8	0.141	1.37	0.71 to 2.67	0.343
Employed	Ref			Ref		
*Type of family*						
Nuclear	0.8	0.4 to 1.3	0.426	0.74	0.41 to 1.33	0.322
Joint	Ref			Ref		
*Monthly family income (in rupees)*						
Less than 15,000	0.9	0.5 to 1.6	0.908	0.82	0.41 to 1.67	0.602
15,000 and above	Ref			Ref		
*Presence of health problems during the current pregnancy*						
Yes	1.8	1.0 to 3.2	0.024^∗^	1.63	0.89 to 2.98	0.107
No	Ref			Ref		
*Thyroid problem*						
Yes	1.15	0.4 to 2.6	0.740	0.92	0.35 to 2.38	0.869
No	Ref			Ref		
*History of psychiatric illness in the past*						
Yes	3.6	0.5 to 26.2	0.202	5.32	0.58 to 48.7	0.139
No	Ref			Ref		
*Alcoholism in a spouse*						
Yes	0.5	0.2 to 1.4	0.219	0.44	0.14 to 1.32	0.144
No	Ref			Ref		
*Parity*						
Primigravida	0.8	0.4 to 1.4	0.481	1.13	0.33 to 3.81	0.843
Multigravida	Ref			Ref		
*Previous history of miscarriage*						
Yes	1.5	0.8 to 2.7	0.177	1.56	0.66 to 3.70	0.308
No	Ref			Ref		

^∗^Significant.

## Data Availability

The data used to support the findings of this study are available from the first author upon request.

## Abbreviations

AND: Antenatal depression
EPDS: Edinburgh Postnatal Depression Scale
COR: Crude odds ratio
AOR: Adjusted odds ratio
CI: Confidence interval.
